# Racial/Ethnic Disparities in Tobacco Product Use Among Middle and High School Students — United States, 2014–2017

**DOI:** 10.15585/mmwr.mm6734a3

**Published:** 2018-08-31

**Authors:** Satomi Odani, Brian S. Armour, Israel T. Agaku

**Affiliations:** 1 Office on Smoking and Health, National Center for Chronic Disease Prevention and Health Promotion, CDC.

## Abstract

During the past few decades, wide disparities in tobacco product use have been documented among the largest racial/ethnic groups in the United States (*1*,*2*); however, little is known about tobacco product use among youths from racial/ethnic groups other than whites, blacks, and Hispanics. Surveillance reports typically aggregate these racial/ethnic minorities into a single category because of small sample sizes (*3*). To assess tobacco product use among U.S. middle and high school students from seven racial/ethnic groups (non-Hispanic whites [whites], non-Hispanic blacks [blacks], Hispanics, non-Hispanic Asians [Asians], non-Hispanic American Indian/Alaska natives [AI/ANs], non-Hispanic Native Hawaiians/Other Pacific Islanders [NHOPIs], and non-Hispanic multiracial persons [multiracial]), CDC analyzed pooled data from the 2014–2017 National Youth Tobacco Surveys (NYTS). Prevalence of ever (≥1 time in lifetime) and current (≥1 time in past 30 days) use of seven tobacco products (cigarettes, cigars, smokeless tobacco, electronic cigarettes [e-cigarettes], hookahs, pipes, and bidis) was assessed; any tobacco product use was defined as use of one or more tobacco products, including hand-rolled cigarettes. During 2014–2017, ever-use of any tobacco product among U.S. middle and high school students was as follows: NHOPIs (45.1%), AI/ANs (43.8%), multiracial persons (38.2%), Hispanics (35.1%), blacks (32.3%), whites (32.0%), and Asians (16.3%). Current use of any tobacco product was as follows: NHOPIs (23.4%), AI/ANs (20.6%), multiracial persons (16.5%), whites (15.3%), Hispanics (14.6%), blacks (11.5%), and Asians (5.0%). Among black middle and high school students, cigars were the most common product currently used, whereas e-cigarettes were the most commonly used product for all other racial/ethnic groups. Comprehensive and sustained implementation of evidence-based, population-level tobacco control interventions could reduce prevalence and disparities in tobacco product use among U.S. youths.

NYTS is a cross-sectional, voluntary, school-based, paper-and-pencil questionnaire administered to U.S. middle (grades 6–8) and high (grades 9–12) school students. A three-stage cluster sampling procedure was used to generate a nationally representative sample of U.S. students attending public and private schools.* For these analyses, data were pooled across four cycles of NYTS (2014–2017) to allow sufficient sample size to assess tobacco product use among each of the racial/ethnic groups. Averaged annual sample size and response rate were 19,566 and 69.1%, respectively.^†^

Participants were asked about ever^§^ and current^¶^ use of seven tobacco product types: cigarettes, cigars (cigars, cigarillos, and little cigars), smokeless tobacco products (chewing tobacco, snuff, dip, snus, and dissolvable tobacco), e-cigarettes, hookahs, pipes, and bidis. Use of hand-rolled cigarettes was also assessed in the survey, but is not reported separately as a distinct tobacco product type because the majority (88.7%) of persons who reported smoking hand-rolled cigarettes also smoked regular cigarettes. Any tobacco product use was defined as use of ≥1 tobacco product types, including hand-rolled cigarettes.

Data were weighted to adjust for differential nonresponse and to yield nationally representative estimates. Pooled sample sizes for each racial/ethnic group during 2014–2017 included 32,358 whites, 11,664 blacks, 21,337 Hispanics, 3,321 Asians, 1,213 AI/ANs, 456 NHOPIs, and 4,106 multiracial persons. Prevalence was calculated overall and by race/ethnicity; within each racial/ethnic group, prevalence was further stratified by sex and school level (middle or high school). Comparisons between and within each racial/ethnic group were performed with chi-squared tests, with statistical significance defined as p<0.05. Estimates with relative standard errors ≥30% were suppressed. Differences in any tobacco product use across groups were assessed using Poisson regression models adjusted for sex and school level. Adjusted prevalence ratios (aPRs) with 95% confidence intervals (CIs) were calculated, with the group with the lowest prevalence (Asians) serving as the referent group.

## Ever-Use of Tobacco Products

During 2014–2017, ever-use of any tobacco product among U.S. middle and high school students was highest among NHOPIs (45.1%) and AI/ANs (43.8%), and did not significantly differ between these groups. Compared with these two groups, prevalence of ever-use of any tobacco product was significantly lower among multiracial persons (38.2%), Hispanics (35.1%), blacks (32.3%), whites (32.0%), and Asians (16.3%) (Table 1). Males reported significantly higher ever-use of any tobacco product than did females among whites (males: 34.6% versus females: 29.5%); Hispanics (36.4% versus 33.8%); and Asians (18.5% versus 13.8%); no significant gender differences were observed for the other racial/ethnic groups. Across all racial/ethnic groups, ever-use of any tobacco product was significantly higher among high school students than among middle school students. By specific product, e-cigarettes were the most commonly ever-used tobacco product, both overall (22.9%) and among all racial/ethnic groups except black and AI/AN students, who reported higher ever-use of cigars (19.9%) and cigarettes (31.4%), respectively (Table 1).

**TABLE 1:** Prevalence of ever use* of tobacco products among middle and high school students, by race/ethnicity,^†^ sex, and school level — National Youth Tobacco Survey, United States, 2014–2017^§^

Characteristic	Any tobacco^¶^	Electronic cigarettes	Cigarettes	Cigars (cigars/cigarillos/little cigars)	Hookah	Smokeless tobacco**	Pipe tobacco	Bidis
	% (95% CI)	% (95% CI)	% (95% CI)	% (95% CI)	% (95% CI)	% (95% CI)	% (95% CI)	% (95% CI)
**All MS and HS students**	32.5 (32.1–32.9)	22.9 (22.5–23.3)	20.2 (19.9–20.6)	16.1 (15.7–16.4)	11.5 (11.2–11.7)	9.2 (9.0–9.5)	2.7 (2.5–2.8)	1.3 (1.2–1.4)
**White**								
Overall	32.0 (31.4–32.7)	23.9 (23.3–24.4)	19.9 (19.4–20.5)	15.8 (15.3–16.3)	10.5 (10.1–10.9)	11.6 (11.2–12.1)	3.0 (2.8–3.3)	1.3 (1.1–1.4)
Male	34.6 (33.7–35.5)^††^	25.6 (24.8–26.5)^††^	20.8 (20.0–21.6)^††^	20.0 (19.2–20.7)^††^	10.8 (10.2–11.4)	16.8 (16.1–17.5)^††^	4.1 (3.8–4.5)^††^	1.7 (1.5–2.0)^††^
Female	29.5 (28.5–30.4)	22.0 (21.2–22.9)	19.0 (18.2–19.7)	11.5 (10.9–12.1)	10.2 (9.7–10.8)	6.4 (5.9–6.9)	1.9 (1.6–2.1)	0.7 (0.6–0.9)
MS	14.9 (14.1–15.7)^††^	10.4 (9.7–11.0)^††^	8.5 (7.9–9.2)^††^	4.2 (3.8–4.7)^††^	2.6 (2.3–2.9)^††^	4.8 (4.3–5.2)^††^	0.9 (0.7–1.1)^††^	0.4 (0.3–0.6)^††^
HS	44.7 (43.8–45.6)	33.8 (33.0–34.7)	28.3 (27.5–29.1)	24.4 (23.6–25.2)	16.4 (15.7–17.0)	16.7 (16.0–17.4)	4.6 (4.2–5.0)	1.9 (1.6–2.1)
**Black**								
Overall	32.3 (31.2–33.5)	16.0 (15.1–16.8)	18.0 (17.1–18.9)	19.9 (18.9–20.8)	9.5 (8.8–10.2)	3.2 (2.8–3.6)	1.0 (0.7–1.2)	1.0 (0.7–1.2)
Male	32.3 (30.7–34.0)	17.6 (16.3–18.9)^††^	18.9 (17.5–20.2)	20.5 (19.1–21.8)	8.6 (7.7–9.6)^††^	4.4 (3.7–5.1)^††^	1.2 (0.8–1.5)	1.0 (0.7–1.4)
Female	32.3 (30.7–33.9)	14.3 (13.1–15.4)	17.1 (15.8–18.4)	19.3 (17.9–20.6)	10.5 (9.5–11.4)	1.8 (1.4–2.3)	0.8 (0.5–1.0)	0.9 (0.6–1.2)
MS	19.0 (17.5–20.5)^††^	10.6 (9.5–11.7)^††^	10.7 (9.6–11.9)^††^	9.0 (7.9–10.0)^††^	4.5 (3.7–5.3)^††^	2.1 (1.6–2.6)^††^	0.8 (0.5–1.0)	0.5 (0.3–0.7)^††^
HS	42.3 (40.7–43.9)	19.9 (18.6–21.1)	23.3 (22.0–24.6)	27.9 (26.5–29.3)	13.2 (12.2–14.3)	3.9 (3.2–4.5)	1.1 (0.8–1.5)	1.3 (1.0–1.7)
**Hispanic**								
Overall	35.1 (34.3–36.0)	26.0 (25.2–26.7)	22.2 (21.5–22.9)	15.7 (15.1–16.4)	15.0 (14.4–15.6)	7.7 (7.2–8.1)	2.7 (2.4–2.9)	1.7 (1.5–1.9)
Male	36.4 (35.2–37.6)^††^	28.1 (27.0–29.1)^††^	23.4 (22.3–24.4)^††^	17.6 (16.7–18.5)^††^	14.4 (13.5–15.2)	9.7 (9.0–10.4)^††^	3.2 (2.8–3.7)^††^	1.8 (1.5–2.1)
Female	33.8 (32.6–35.0)	23.9 (22.8–24.9)	20.9 (19.9–21.9)	13.8 (13.0–14.7)	15.5 (14.6–16.4)	5.6 (5.0–6.1)	2.0 (1.7–2.3)	1.5 (1.2–1.9)
MS	20.8 (19.7–22.0)^††^	15.6 (14.7–16.6)^††^	12.4 (11.5–13.3)^††^	7.9 (7.1–8.6)^††^	7.9 (7.1–8.6)^††^	4.7 (4.1–5.3)^††^	1.6 (1.3–1.9)^††^	1.0 (0.8–1.3)^††^
HS	46.7 (45.5–47.8)	34.4 (33.3–35.4)	30.1 (29.1–31.1)	22.0 (21.1–22.9)	20.6 (19.7–21.5)	9.8 (9.1–10.5)	3.4 (3.0–3.8)	2.1 (1.8–2.4)
**Asian**								
Overall	16.3 (14.6–17.9)	11.0 (9.6–12.4)	10.3 (9.0–11.6)	4.7 (3.8–5.5)	5.7 (4.8–6.6)	3.1 (2.3–3.9)	0.8 (0.5–1.2)	0.4 (0.2–0.6)
Male	18.5 (16.1–20.9)^††^	12.2 (10.3–14.2)	12.1 (10.2–14.1)^††^	5.8 (4.5–7.0)^††^	5.4 (4.2–6.6)	3.9 (2.8–5.1)^††^	1.0 (0.5–1.5)	—^§§^
Female	13.8 (11.7–16.0)	9.6 (7.7–11.5)	8.2 (6.5–9.9)	3.5 (2.4–4.6)	6.0 (4.6–7.5)	2.2 (1.2–3.2)	—^§§^	—^§§^
MS	5.4 (3.8–6.9)^††^	4.6 (3.2–6.1)^††^	4.8 (3.2–6.4)^††^	2.1 (1.1–3.1)^††^	1.6 (0.9–2.4)^††^	1.7 (0.8–2.7)^††^	—^§§^	—^§§^
HS	24.4 (21.9–26.9)	15.7 (13.6–17.8)	14.2 (12.3–16.2)	6.6 (5.3–7.8)	8.7 (7.2–10.1)	4.1 (3.0–5.2)	1.2 (0.6–1.7)	—^§§^
**AI/AN**								
Overall	43.8 (39.2–48.4)	29.8 (25.7–33.9)	31.4 (27.2–35.7)	23.1 (19.2–26.9)	14.2 (11.1–17.3)	18.6 (14.6–22.5)	5.3 (3.0–7.6)	2.0 (1.1–3.0)
Male	43.7 (37.7–49.8)	31.4 (26.0–36.7)	32.1 (26.7–37.4)	25.0 (19.9–30.1)	12.7 (9.0–16.3)	23.5 (17.9–29.1)^††^	8.5 (4.5–12.6)	—^§§^
Female	44.2 (37.0–51.4)	27.9 (21.5–34.4)	31.1 (24.4–37.9)	20.9 (15.0–26.9)	16.3 (11.0–21.6)	12.3 (7.0–17.6)	—^§§^	—^§§^
MS	28.5 (22.2–34.7)^††^	18.6 (13.5–23.7)^††^	20.8 (15.3–26.3)^††^	12.0 (7.5–16.6)^††^	6.6 (3.8–9.3)^††^	13.1 (8.2–17.9)^††^	—^§§^	—^§§^
HS	62.8 (56.5–69.2)^††^	43.2 (36.9–49.5)	44.4 (38.1–50.7)	36.1 (29.9–42.3)	23.5 (17.8–29.1)	25.4 (19.2–31.7)	7.9 (4.3–11.4)	3.1 (1.3–4.9)
**NHOPI**								
Overall	45.1 (38.4–51.8)	34.1 (28.0–40.2)	29.4 (23.4–35.4)	22.4 (17.1–27.8)	20.6 (15.5–25.6)	13.1 (8.9–17.4)	4.7 (2.2–7.3)	—^§§^
Male	49.7 (40.9–58.5)	37.3 (29.2–45.5)	33.2 (25.1–41.4)	28.0 (20.4–35.6)	21.6 (14.7–28.5)	20.4 (13.3–27.5)	—^§§^	—^§§^
Female	40.8 (30.7–50.9)	31.0 (21.8–40.2)	25.2 (16.2–34.2)	15.2 (7.6–22.8)	19.1 (11.6–26.6)	—^§§^	—^§§^	—^§§^
MS	23.5 (15.9–31.1)^††^	18.7 (12.0–25.4)^††^	14.8 (8.6–21.1)^††^	10.7 (5.2–16.2)^††^	10.5 (4.7–16.2)^††^	8.3 (3.5–13.0)	—^§§^	—^§§^
HS	60.7 (52.4–69.0)	44.9 (36.6–53.2)	39.6 (31.2–48.0)	30.6 (22.8–38.4)	27.5 (20.1–34.8)	16.6 (10.3–22.9)	—^§§^	—^§§^
**Multiracial**								
Overall	38.2 (36.1–40.2)	26.6 (24.8–28.5)	24.9 (23.1–26.7)	18.5 (16.9–20.1)	14.3 (12.8–15.8)	10.1 (8.8–11.5)	4.5 (3.4–5.6)	1.6 (1.1–2.1)
Male	38.8 (35.8–41.8)	28.2 (25.5–30.8)	25.2 (22.7–27.7)	21.5 (19.1–23.9)^††^	14.3 (12.2–16.4)	14.0 (11.9–16.2)^††^	6.1 (4.5–7.7)^††^	1.8 (1.1–2.6)
Female	37.6 (34.7–40.4)	25.1 (22.6–27.7)	24.6 (22.0–27.2)	15.6 (13.6–17.7)	14.3 (12.2–16.4)	6.7 (4.9–8.4)	3.2 (1.6–4.7)	1.4 (0.7–2.0)
MS	24.5 (21.6–27.5)^††^	15.8 (13.3–18.3)^††^	15.2 (12.7–17.7)^††^	8.7 (6.9–10.5)^††^	5.7 (3.8–7.6)^††^	6.8 (4.8–8.8)^††^	—^§§^	0.9 (0.4–1.4)^††^
HS	48.4 (45.7–51.1)	34.6 (32.1–37.2)	32.2 (29.7–34.6)	25.8 (23.5–28.1)	20.7 (18.6–22.9)	12.6 (10.8–14.5)	6.1 (4.6–7.6)	2.1 (1.3–2.9)

**Abbreviations:** AI/AN = American Indians/Alaska Natives; CI = confidence interval; HS = high school; MS = middle school; NHOPI = Native Hawaiian/Other Pacific Islander.

* Respondents were asked whether they had ever used/smoked the respective tobacco products, or asked “Which of the following tobacco products have you ever tried, even just one time?,” depending on the type of products and survey years. Those who provided an affirmative response or specified their product(s) from the product list provided were classified as ever tobacco product users.

^†^ All racial/ethnic groups assessed are non-Hispanic unless otherwise specified

^§^ Data were pooled across four cycles of National Youth Tobacco Surveys (2014–2017) to increase precision of estimates among racial/ethnic minorities. Pooled sample sizes for each racial/ethnic group during 2014–2017 were 32,358 whites; 11,664 blacks; 21,337 Hispanics; 3,321 Asians; 1,213 AI/ANs; 456 NHOPIs; and 4,106 multiracial persons.

^¶^ Any tobacco product use was defined as use of one or more tobacco product types, including hand-rolled cigarettes. Smokers of hand-rolled cigarettes are not reported separately because the vast majority (88.7%) of these persons reported ever smoking regular cigarettes.

** Chewing tobacco/snuff/dip/snus/dissolvable tobacco.

^††^ Prevalence significantly different (p<0.05) within demographic subgroups (male versus female; MS versus HS).

^§§^ Estimates not presented because relative standard error ≥30%.

## Current Use of Tobacco Products

Current use of any tobacco product was highest among NHOPIs (23.4%) and AI/ANs (20.6%), followed by multiracial persons (16.5%); whites (15.3%); Hispanics (14.6%), blacks (11.5%), and Asians (5.0%) (Table 2). Compared with Asians, and controlling for sex and school level, current use of any tobacco product was significantly higher among blacks (aPR = 2.33; 95% CI = 1.91–2.82), Hispanics (2.97; 2.46–3.58), whites (3.08; 2.56–3.71), multiracial persons 3.37; 2.74–4.13), NHOPIs (4.61; 3.44–6.19), and AI/ANs (4.84; 3.78–6.21) (Figure). Males reported significantly higher current use of any tobacco product than did females among whites (males: 18.0% versus females: 12.6%), Hispanics (15.6% versus 13.5%), NHOPIs (29.6% versus 16.8%), and multiracial persons (19.5% versus 13.8%); no significant gender differences were observed for the other racial/ethnic groups. Across all racial/ethnic groups, current use of any tobacco product was significantly higher among high school students than among middle school students. E-cigarettes were the most common currently used tobacco product overall (9.2%) and among all racial/ethnic groups except black students (Table 2), among whom the most common currently used product was cigars (6.7%), followed by e-cigarettes (5.1%).

**TABLE 2:** Prevalence of current use* of tobacco products among middle and high school students, by race/ethnicity,^†^ sex, and school level — National Youth Tobacco Survey, United States, 2014–2017^§^

Characteristic	Any tobacco^¶^	Electronic cigarettes	Cigarettes	Cigars (cigars/cigarillos/little cigars)	Hookah	Smokeless tobacco**	Pipe tobacco	Bidis
	% (95% CI)	% (95% CI)	% (95% CI)	% (95% CI)	% (95% CI)	% (95% CI)	% (95% CI)	% (95% CI)
**All MS and HS students**	14.3 (14–14.6)	9.2 (9.0–9.5)	5.8 (5.6–6.0)	5.3 (5.1–5.5)	4.4 (4.2–4.5)	4.0 (3.8–4.2)	0.9 (0.8–1.0)	0.6 (0.5–0.6)
**White**								
Overall	15.3 (14.8–15.8)	10.2 (9.8–10.6)	6.6 (6.3–6.9)	5.2 (4.9–5.5)	3.7 (3.5–4.0)	5.0 (4.7–5.3)	0.9 (0.7–1.0)	0.4 (0.3–0.5)
Male	18.0 (17.3–18.7)^††^	11.7 (11.1–12.3)^††^	7.0 (6.6–7.5)^††^	7.2 (6.7–7.7)^††^	3.8 (3.5–4.2)	7.8 (7.3–8.4)^††^	1.2 (1.0–1.4)^††^	0.6 (0.4–0.8)^††^
Female	12.6 (12–13.3)	8.6 (8.1–9.2)	6.1 (5.6–6.6)	3.2 (2.9–3.5)	3.6 (3.3–4.0)	2.2 (1.8–2.5)	0.5 (0.4–0.7)	0.2 (0.2–0.3)
MS	4.9 (4.4–5.3)^††^	3.5 (3.1–3.9)^††^	1.9 (1.6–2.2)^††^	1.2 (1.0–1.5)^††^	1.1 (0.8–1.3)^††^	1.6 (1.3–1.9)^††^	0.4 (0.2–0.5)^††^	0.2 (0.1–0.4)^††^
HS	23.0 (22.2–23.8)	15.1 (14.5–15.7)	10.0 (9.5–10.6)	8.2 (7.7–8.6)	5.7 (5.3–6.1)	7.5 (7.1–8.0)	1.2 (1.0–1.4)	0.6 (0.4–0.7)
**Black**								
Overall	11.5 (10.7–12.3)	5.1 (4.6–5.7)	3.3 (2.8–3.7)	6.7 (6.1–7.3)	3.5 (3.1–4.0)	1.5 (1.2–1.7)	0.6 (0.4–0.7)	0.6 (0.4–0.8)
Male	12.2 (11.0–13.4)	5.8 (5.0–6.5)^††^	4.1 (3.4–4.8)^††^	7.6 (6.6–8.5)^††^	3.3 (2.6–3.9)	2.0 (1.5–2.4)^††^	0.7 (0.4–1.0)	0.6 (0.3–0.8)
Female	10.8 (9.7–11.9)	4.4 (3.7–5.1)	2.3 (1.8–2.8)	5.9 (5.2–6.7)	3.7 (3.1–4.4)	0.9 (0.5–1.2)	0.4 (0.2–0.6)	0.6 (0.3–0.9)
MS	5.7 (4.9–6.6)^††^	3.5 (2.8–4.1)^††^	1.7 (1.2–2.1)^††^	2.5 (2.0–3.1)^††^	1.8 (1.3–2.3)^††^	1.2 (0.7–1.6)	0.5 (0.2–0.8)	0.4 (0.2–0.6)
HS	15.8 (14.6–17.0)	6.3 (5.5–7.1)	4.3 (3.7–5.0)	9.9 (8.9–10.8)	4.8 (4.1–5.4)	1.6 (1.2–2.0)	0.6 (0.4–0.8)	0.8 (0.5–1.0)
**Hispanic**								
Overall	14.6 (14.0–15.2)	9.9 (9.4–10.4)	5.7 (5.3–6.1)	5.3 (4.9–5.7)	6.2 (5.8–6.6)	3.5 (3.2–3.8)	1.2 (1.0–1.4)	0.8 (0.7–1.0)
Male	15.6 (14.7–16.5)^††^	11.3 (10.6–12.1)^††^	6.3 (5.7–6.9)^††^	5.9 (5.4–6.5)^††^	5.7 (5.2–6.2)^††^	4.6 (4.1–5.2)^††^	1.4 (1.1–1.6)	0.9 (0.7–1.1)
Female	13.5 (12.6–14.3)	8.5 (7.8–9.1)	5.0 (4.5–5.6)	4.6 (4.1–5.1)	6.6 (6.0–7.3)	2.2 (1.9–2.6)	1.0 (0.8–1.2)	0.7 (0.5–0.9)
MS	8.2 (7.4–9.0)^††^	6.0 (5.4–6.6)^††^	3.1 (2.6–3.6)^††^	2.6 (2.2–2.9)^††^	3.8 (3.2–4.3)^††^	2.5 (2.1–3.0)^††^	0.9 (0.6–1.1)^††^	0.6 (0.4–0.8)^††^
HS	19.6 (18.7–20.5)	12.9 (12.2–13.7)	7.6 (7.0–8.2)	7.3 (6.7–7.8)	8.0 (7.4–8.6)	4.0 (3.5–4.4)	1.4 (1.1–1.6)	0.9 (0.7–1.1)
**Asian**								
Overall	5.0 (4.1–5.9)	3.6 (2.9–4.4)	2.0 (1.4–2.5)	1.1 (0.7–1.6)	1.9 (1.4–2.4)	0.7 (0.4–1.0)	—^§§^	—^§§^
Male	5.8 (4.5–7.2)	4.1 (3.0–5.3)	2.0 (1.2–2.7)	1.4 (0.7–2.1)	1.8 (1.1–2.5)	0.9 (0.4–1.3)	—^§§^	—^§§^
Female	4.1 (2.9–5.3)	3.1 (2.0–4.2)	2.0 (1.1–2.8)	0.9 (0.4–1.3)	2.0 (1.2–2.8)	—^§§^	—^§§^	—^§§^
MS	1.8 (0.9–2.6)^††^	1.5 (0.8–2.2)^††^	—^§§^	—^§§^	—^§§^	—^§§^	—^§§^	—^§§^
HS	7.4 (5.9–8.8)	5.2 (4.0–6.5)	2.5 (1.7–3.3)	1.5 (0.9–2.2)	2.8 (2.0–3.6)	0.7 (0.4–1.1)	—^§§^	—^§§^
**AI/AN**								
Overall	20.6 (17.0–24.3)	12.7 (10.1–15.3)	10.3 (7.6–13.1)	6.8 (4.8–8.7)	5.9 (4.1–7.8)	7.7 (5.0–10.3)	—^§§^	—^§§^
Male	21.8 (17.1–26.6)	14.5 (10.8–18.2)	10.0 (6.7–13.3)	7.8 (5.0–10.7)	4.9 (2.7–7.1)	8.1 (5.1–11.1)	—^§§^	—^§§^
Female	19.1 (13.3–24.9)	10.4 (6.8–14.1)	10.9 (6.1–15.6)	5.6 (3.0–8.1)	7.2 (4.0–10.4)	6.9 (2.2–11.5)	—^§§^	—^§§^
MS	9.0 (6.2–11.9)^††^	6.4 (3.8–8.9)^††^	5.2 (2.6–7.8)^††^	3.0 (1.5–4.6)^††^	^§§^	3.3 (1.7–5.0)^††^	—^§§^	—^§§^
HS	35.4 (28.8–42.1)	20.1 (15.5–24.8)	16.7 (11.6–21.9)	11.4 (7.5–15.2)	9.9 (6.5–13.4)	13.0 (7.7–18.3)	—^§§^	—^§§^
**NHOPI**								
Overall	23.4 (17.6–29.2)	18.0 (13.1–22.9)	9.5 (5.4–13.6)	11.1 (7.0–15.1)	9.5 (6.1–12.9)	7.3 (4.2–10.4)	—^§§^	—^§§^
Male	29.6 (21.0–38.3)^††^	25.7 (17.9–33.6)^††^	13.1 (6.9–19.3)	15.8 (9.1–22.6)	10.9 (5.7–16.1)	12.0 (6.3–17.7)	—^§§^	—^§§^
Female	16.8 (9.4–24.2)	9.5 (4.4–14.5)	—^§§^	—^§§^	7.3 (3.1–11.5)	—^§§^	—^§§^	—^§§^
MS	10.1 (4.7–15.5)^††^	10.8 (5.2–16.4)^††^	—^§§^	—^§§^	—^§§^	—^§§^	—^§§^	—^§§^
HS	32.8 (24.3–41.3)	23.1 (15.9–30.3)	12.5 (6.3–18.7)	14.2 (8.1–20.2)	11.1 (6.4–15.8)	8.5 (4.1–12.9)	—^§§^	—^§§^
**Multiracial**								
Overall	16.5 (15.0–18.0)	10.6 (9.3–11.9)	6.6 (5.6–7.7)	6.0 (5.1–6.9)	5.8 (4.9–6.8)	4.0 (3.2–4.9)	1.5 (1.0–2.0)	—^§§^
Male	19.5 (17.0–21.9)^††^	12.5 (10.4–14.5)^††^	8.0 (6.4–9.6)^††^	7.7 (6.1–9.3)^††^	6.1 (4.7–7.6)	6.9 (5.3–8.6)^††^	2.0 (1.2–2.9)	—^§§^
Female	13.8 (12.0–15.7)	8.9 (7.3–10.5)	5.4 (4.1–6.8)	4.6 (3.6–5.6)	5.6 (4.4–6.9)	1.5 (0.9–2.1)	1.0 (0.4–1.6)	—^§§^
MS	7.6 (6.0–9.1)^††^	5.3 (4.0–6.6)^††^	2.6 (1.8–3.4)^††^	2.2 (1.5–3.0)^††^	2.4 (1.5–3.2)^††^	2.2 (1.4–3.1)^††^	—^§§^	—^§§^
HS	23.1 (20.8–25.4)	14.5 (12.5–16.5)	9.6 (8.0–11.3)	8.8 (7.4–10.3)	8.4 (6.9–9.9)	5.4 (4.1–6.7)	2.0 (1.2–2.7)	—^§§^

**Abbreviations:** AI/AN = American Indians/Alaska Natives; CI = confidence interval; HS = high school; MS = middle school; NHOPI = Native Hawaiian/Other Pacific Islander.

* Respondents were asked on how many days in the past 30 days they used/smoked the respective tobacco products, or asked “In the past 30 days, which of the following products have you used on at least one day?,” depending on the type of products and survey years. Those who answered ≥1 day or specified their product(s) from the product list provided were classified as current tobacco product users.

^†^ All racial/ethnic groups assessed are non-Hispanic, unless otherwise specified.

^§^ Data were pooled across four cycles of National Youth Tobacco Surveys (2014–2017) to increase precision of estimates among racial/ethnic minorities. Pooled sample sizes for each racial/ethnic group during 2014–2017 were 32,358 whites; 11,664 blacks; 21,337 Hispanics; 3,321 Asians; 1,213 AI/ANs; 456 NHOPIs; and 4,106 multiracial persons.

^¶^ Any tobacco product use was defined as use of one or more tobacco product types, including hand-rolled cigarettes. Smokers of hand-rolled cigarettes are not reported separately because the vast majority (88.7%) of these individuals reported ever smoking regular cigarettes.

** Chewing tobacco/snuff/dip/snus/dissolvable tobacco.

^††^ Prevalence significantly different within demographic subgroups (male versus female; MS versus HS) (p<0.05).

^§§^ Estimates not presented because relative standard error ≥30%.

**FIGURE:**
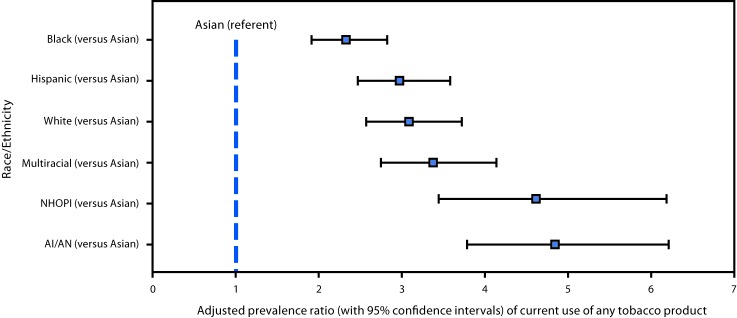
Adjusted prevalence ratios* of current use of any tobacco product^†^ among middle and high school students — National Youth Tobacco Survey, United States, 2014–2017^§^^,^^¶^ **Abbreviations:** AI/AN = American Indians/Alaska Natives; NHOPI = Native Hawaiian/Other Pacific Islander. * Adjusted prevalence ratios and 95% confidence intervals (presented as whiskers) were obtained using Poisson regression models adjusted for sex and school level, with the group with the lowest prevalence of current use of any tobacco product (Asians, 5.0%) serving as the referent. Among other racial and ethnic groups, prevalence was NHOPIs 23.4%; AI/ANs 20.6%; multiracial 16.5%; whites 15.3%; Hispanics 14.6%; and blacks 11.5%. ^†^ Current (≥1 time in the past 30 days) use of any tobacco product was defined as current use of one or more tobacco product types, including cigarettes, cigars (including cigarillos and little cigars), smokeless tobacco (including chewing tobacco, snuff, dip, snus, and dissolvable tobacco), electronic cigarettes, hookahs, pipes, bidis, and hand-rolled cigarettes. ^§^ Data were pooled across four cycles of NYTS (2014–2017) to increase precision of estimates among racial and ethnic minorities. Pooled sample sizes for each racial/ethnic group during 2014–2017 were 32,358 whites; 11,664 blacks; 21,337 Hispanics; 3,321 Asians; 1,213 AI/ANs; 456 NHOPIs; and 4,106 multiracial. ^¶^ All racial/ethnic groups assessed are non-Hispanic, unless otherwise specified.

## Discussion

Marked disparities in tobacco product use exist among U.S. youths by race/ethnicity. Tobacco product use is higher among NHOPIs and AI/ANs, with nearly one in two NHOPI (45.1%) and AI/AN (43.8%) youths reporting ever using at least one tobacco product. Early exposure to nicotine during adolescence can lead to stronger addiction to tobacco products (*2*), and tobacco product experimentation is a critical step in developing dependence (*4*). Given that most adult smokers first try cigarettes before age 18 years, and that progression from occasional to daily smoking typically occurs during early adulthood (*2*), these disparities among youths might contribute to the higher rates of tobacco product use among adults from these racial/ethnic groups (*1*,*5*).

Use of specific tobacco products varied by race/ethnicity. Ever-use was highest for cigarettes among AI/AN students (31.4%), highest for cigars among black students (19.9%), and highest for e-cigarettes among all other racial/ethnic groups. E-cigarettes were the most common currently used tobacco product among youths overall (9.2%) and all racial/ethnic groups except blacks, among whom cigar smoking was most prevalent. Given that cigar smoking has historically been higher among black adults than other racial/ethnic groups (*6*,*7*), these findings suggest distinct acculturation and social norms regarding tobacco use across racial/ethnic groups.

Observed disparities in tobacco product use might also be attributable to racial/ethnic variations in targeted tobacco industry advertising, marketing, and promotional activities (*1*,*2*,*8*). For example, some cigarettes have been promoted using tribal icons and logos to attract AI/AN persons (*1*). In addition, mentholated and other flavored tobacco products have been heavily promoted to certain racial/ethnic minority populations, including black communities (*9*). Flavored additives can mask the harshness of tobacco products, which might make it easier for nonusers to try their first tobacco product (*9*).

The findings in this report are subject to at least three limitations. First, tobacco product use was self-reported and might have been subject to recall and social desirability bias. Second, small sample sizes of some subgroups within the assessed racial/ethnic categories resulted in imprecise estimates that could not be reported. Finally, these analyses used pooled data across 4 years, and therefore do not reflect possible secular trends in prevalence and disparities in youth tobacco product use.

Evidence-based strategies that have been proven to reduce youth tobacco use include tobacco product price increases, policies that protect persons from secondhand smoke exposure from combustible tobacco and e-cigarette aerosol, advertising and promotion restrictions, national public education campaigns, and strategies to reduce youth access to flavored tobacco products, including menthol (*1*,*2*,*9*). In addition, states and communities have worked to reduce youth tobacco use by raising the minimum age for sale of tobacco products to 21 years (*10*). As of August 2018, six states (California, Hawaii, Maine, Massachusetts, New Jersey, and Oregon) and several hundred localities have raised the minimum age of tobacco product sales to 21 years.** Ensuring that these interventions reach all population groups, coupled with targeted strategies that acknowledge sociocultural dimensions of tobacco use among racial/ethnic groups, could reduce tobacco product use and tobacco-related disparities among U.S. youths (*2*,*5*).

**Summary**

**What is known about this topic?**

Wide disparities in tobacco product use have been documented among the largest U.S. racial/ethnic groups; however, apart from the three most populous groups (non-Hispanic whites, non-Hispanic blacks, and Hispanics), little is known about tobacco use among youths from other racial/ethnic groups.

**What is added by this report?**

During 2014–2017, ever-use and current use of any tobacco product among U.S. middle and high school students were highest among Native Hawaiians/Other Pacific Islanders and American Indians/Alaska Natives and lowest among Asians.

**What are the implications for public health practice?**

Comprehensive and sustained implementation of evidence-based, population-level tobacco control interventions could reduce prevalence of and disparities in tobacco product use among U.S. youths.

## Footnotes

* https://www.cdc.gov/tobacco/data_statistics/surveys/nyts/index.htm.

† Annual sample sizes and response rates were 22,007: 73.3% (2014); 17,711: 63.4% (2015); 20,675: 71.6% (2016); and 17,872: 68.1% (2017).

§ Respondents were asked whether they have ever used/smoked the respective tobacco products, or asked “Which of the following tobacco products have you ever tried, even just one time?,” depending on the type of products and survey years. Those who provided an affirmative response or specified their product(s) from the product list provided were classified as ever tobacco product users.

¶ Respondents were asked on how many days in the past 30 days they used/smoked the respective tobacco products, or asked “In the past 30 days, which of the following products have you used on at least one day?,” depending on the type of products and survey years. Those who answered ≥1 day or specified their product(s) from the product list provided were classified as current tobacco product users.

** https://tobacco21.org/.
